# Recipient Selection and Outcomes After Out‐of‐Sequence Kidney Allocation Since Implementation of Kidney Allocation System 250 (KAS250): A Single‐Center Experience

**DOI:** 10.1111/ctr.70647

**Published:** 2026-08-13

**Authors:** Sandesh Parajuli, Glen E. Leverson, Didier Mandelbrot, Dixon B. Kaufman

**Affiliations:** ^1^ Division of Nephrology, Department of Medicine University of Wisconsin School of Medicine and Public Health Madison Wisconsin USA; ^2^ Division of Transplant Surgery, Department of Surgery University of Wisconsin School of Medicine and Public Health Madison Wisconsin USA

**Keywords:** graft survival, KAS250, kidney transplantation, out‐of‐sequence allocation, recipient selection

## Abstract

**Introduction:**

Allocation out of sequence (AOOS) of kidneys has increased after KAS250 and raised concerns regarding transparency and equitable recipient selection. Less is known about recipient selection and outcomes at individual centers.

**Methods:**

We performed a single‐center retrospective study of adult deceased donor kidney transplants at the University of Wisconsin from March 15, 2021, to March 31, 2024. Recipients receiving kidneys classified as AOOS were compared with contemporaneous recipients of non‐AOOS kidneys. Donor and recipient characteristics, delayed graft function, graft function, and patient and graft survival were assessed. Standardized data explaining why kidneys entered AOOS allocation were unavailable.

**Results:**

Among 498 deceased donor kidney transplant recipients, 89 (17.9%) received AOOS classified kidneys. Donor characteristics were largely similar, although kidneys categorized as AOOS had higher terminal creatinine (1.6 vs. 1.2 mg/dl, *p* = 0.03) and longer cold ischemia time (22.9 vs. 20.4 h, *p* = 0.003). AOOS recipients were older, more often preemptive, had shorter waiting times, and were less likely to be retransplant recipients or have cPRA > 20%. Recipient sex and race did not differ. Four‐year adjusted patient survival (aHR 0.85; 95% CI 0.38–1.94; *p* = 0.71) and graft survival (aHR 0.72; 95% CI 0.34–1.54; *p* = 0.40) were not statistically different.

**Conclusion:**

In this single‐center experience, transplantation of kidneys categorized as AOOS was associated with distinct recipient selection, comparable 4‐year outcomes, and without compromising gender/racial balance.

**Abbreviations**
AKI: Acute kidney injury
AOOS: Allocation out of sequence
cPRA: Calculated panel reactive antibody
DDKTRs: Deceased donor kidney transplant recipients
DGF: Delayed graft function
eGFR: Estimated glomerular filtration rate
EPTS: Estimated Post‐Transplant Survival
KAS: Kidney Allocation System
KAS250: KAS based on 250‐nautical‐mile circles
KDPI: Kidney donor profile index
KDRI: Kidney donor risk index
OPO: Organ Procurement Organization
SRTR: Scientific Registry of Transplant Recipients

## 1 Introduction

The severe shortage of deceased donor kidneys limits timely access to transplantation for patients with kidney failure. A central goal of national kidney allocation policy is to balance equity, efficiency, and organ utilization while maintaining acceptable post‐transplant outcomes. Kidney Allocation System 250 (KAS250) implemented on March 15, 2021, expanded allocation through 250‐nautical‐mile circles with the intent of reducing geographic disparities and improving access. Since implementation, deceased donor kidney transplantation has increased, but allocation has also become more logistically complex, with higher offer volume, longer placement times for some kidneys, and persistent concern regarding kidney nonuse [1, 2, 3, 4, 5, 6, 7].

Allocation out of sequence (AOOS) occurs when an organ procurement organization allocates a kidney outside the strict match‐run order after prior sequential allocation efforts have not resulted in timely acceptance. AOOS is generally understood as an accelerated placement process intended to avoid nonuse when placement becomes time‐sensitive. However, AOOS has raised important concerns about transparency, oversight, and whether certain candidates or transplant centers may gain disproportionate access [5, 8, 9, 10, 11].

A major challenge in interpreting AOOS allocation is that AOOS status does not, by itself, define a uniform donor phenotype. Kidneys may enter AOOS allocation for many reasons, including logistical constraints, accumulated cold ischemia time, prior declines, biopsy or perfusion concerns, donor acute kidney injury, anatomical considerations, or a combination of factors. Some of these details are not captured consistently in retrospective center‐level datasets or in registry‐based risk models. Therefore, AOOS should not automatically be equated with marginal donor quality or with a specific category of extended‐criteria kidneys [12, 13, 14].

National analyses have described contemporary AOOS prevalence and identified potential inequities in recipient selection [5, 8, 9, 10]. Less is known about how individual centers select recipients for AOOS kidneys and whether those recipients experience outcomes comparable with recipients of non‐AOOS kidneys. This is an important and distinct question because center‐level recipient selection may affect outcomes independent of donor factors measured in registry data.

The objective of this study was to describe recipient selection and 4‐year outcomes associated with kidney transplantation categorized as AOOS at a single center during the KAS250 era. Specifically, we compared donor characteristics, recipient characteristics, delayed graft function, graft function, and patient and graft survival between recipients of AOOS and non‐AOOS deceased donor kidneys. This study was not designed to determine why individual kidneys entered AOOS allocation or to characterize AOOS practice across transplant centers nationally.

## 2 Methods

This is a single‐center, retrospective study conducted at the University of Wisconsin that included all adult deceased donor kidney transplants between March 15, 2021 (when KAS250 was implemented) and March 31, 2024. We excluded recipients of multi‐renal and non‐renal organ transplants. Living donor kidney transplant recipients were also excluded. The outcome of interest was a comparison of donor organ characteristics, recipient demographics, and patient/graft functional survival rates at 1, 2, 3, and 4 years post‐transplant. Graft function was assessed by serum creatinine and estimated glomerular filtration rate (eGFR) using a race‐free CKD‐EPI eGFR calculator. Outcomes also included delayed graft function (DGF), defined as the need for dialysis within seven days post‐transplant, and death‐censored graft survival. The duration of DGF was defined as the interval from transplant to the last dialysis. Patients were followed until the end of the study analysis in December 2025, graft failure, or death.

Allocation status was categorized as AOOS or non‐AOOS based on the allocation designation available in the transplant record. Standardized information describing why individual kidneys entered AOOS allocation was not available in this retrospective dataset. Therefore, donor biopsy findings, machine perfusion parameters, anatomical considerations, prior offer sequence depth, refusal reasons, and logistical factors could not be systematically analyzed as triggers for AOOS allocation.

This study was approved by the University of Wisconsin Health Sciences Institutional Review Board (IRB protocol number: 2014–1072) and adhered to the Declaration of Helsinki. The clinical and research activities reported were consistent with the Principles of the Declaration of Istanbul on Organ Trafficking and Transplant Tourism. Due to the nature of this study, informed consent specific to this research was not obtained from patients.

### 2.1 Immunosuppressive Protocols

The standard protocol for most kidney transplant recipients at our institution involved induction therapy using either a depleting agent (anti‐thymocyte globulin or alemtuzumab) or a non‐depleting agent (basiliximab), this was followed by a maintenance immunosuppressive regimen of tacrolimus and mycophenolic acid, with or without prednisone. The choice between depleting and non‐depleting agents was based on immunological risk factors. Depleting agents were preferred for patients with pre‐transplant donor‐specific antibodies (DSA), glomerulonephritis as the cause of kidney disease, and those who underwent early steroid withdrawal [15]. Although not formalized in a protocol, patients at risk for DGF generally received a depleting agent based on the transplant surgeon's discretion.

### 2.2 Delayed Graft Function (DGF) Management

Patients with DGF have been followed as outpatients in a dedicated DGF clinic, as previously described [16]. They were either discharged to their homes or a nearby hotel with a support person and were required to attend a clinic visit within 1–3 days post‐discharge. Dialysis was scheduled as needed on the same day in our inpatient dialysis unit.

### 2.3 Statistical Analysis

Continuous data were compared using Student's t‐test, the Wilcoxon rank‐sum test, or Kruskal‐Wallis test, as appropriate, while categorical data were analyzed using Fisher's exact test or chi‐square test. Kaplan‐Meier and Cox proportional hazards analyses were performed to examine survival data. P values ≤ 0.05 were considered statistically significant. Factors associated with outcomes of interest were evaluated using univariate and multivariate analyses.

## 3 Results

Four hundred ninety‐eight adult deceased donor kidney transplant recipients were included during the study period. Eighty‐nine (17.9%) were recipients of kidneys categorized as AOOS (Table 1). Of the 89 AOOS kidneys, 57 (64%) were imported, and the remainder were from the local OPO (WIUW). Among the 57 imported AOOS kidneys, the mean (± SD) distance from our center to the donor hospital was 170 ± 124 nautical miles, with 32 (56.1%) ≤ 150 nautical miles, 18 (31.6%) 150–250 nautical miles, 5 (8.8%) 250–500 nautical miles, and 2 (3.5%) more than 500 nautical miles from our center.

**TABLE 1:** Baseline characteristics.

	Variables	AOOS (*n* = 89)	Non‐AOOS (*n* = 409)	*p*
Donor	Mean age (SD) (yrs) (SD)	43.8 (14.6)	42.6 (15.2)	0.52
Donor	Female (%)	33 (37)	138 (34)	0.38
Donor	Mean body mass index (Kg/m2) (SD)	29.2 (6.5)	29.6 (9.2)	0.57
Donor	Cause of death: Cerebrovascular (%)	12 (13)	77 (19)	0.86
Donor	Terminal serum Creatinine (mg/dl) (SD)	1.6 (1.2)	1.2 (1.2)	0.03
Donor	Mean kidney donor profile index % (SD)	50.9 (24.9)	49.1 (26.5)	0.54
Donor	KDPI > 85% (%)	7 (7.9)	40 (9.8)	0.69
Donor	Donation after circulatory death (%)	43 (48)	176 (43)	0.41
Donor	Right kidney (%) Dual kidney (%) En‐bloc	45 (51) 5 (6) 0	198 (48) 6 (1) 7 (2)	0.08
Donor	Cold ischemia time (hours) (SD)	22.9 (5.5)	20.4 (5.7)	0.003
Immunologic	cPRA > 20% (%)	9 (10)	87 (21)	0.03
Immunologic	Mean HLA mismatch (of 6) (SD)	4.4 (1.4)	4.1 (1.4)	0.09
Immunologic	Previous transplant (%)	5 (6)	67 (16)	0.007
Recipient's Factors	Mean age (yrs) (SD)	58.9 (12.7)	54.8 (13.2)	0.007
Recipient's Factors	Female (%)	26 (29.2)	149 (36.4)	0.22
Recipient's Factors	Race (%) White Black or African American Asian Other/decline	60 (67.4) 17 (19.1) 10 (11.2) 2 (2.3)	281 (68.7) 77 (18.8) 40 (9.8) 11 (2.7)	0.85
Recipient's Factors	Mean body mass index (Kg/m2) (SD)	28.5 (5.3)	29.1 (5.3)	0.36
Recipient's Factors	Causes of ESRD (%) Diabetes Hypertension Glomerulonephritis Polycystic kidney disease Other	26 (29) 24 (27) 11 (12) 7 (14) 21 (24)	111 (27) 77 (19) 73 (18) 49 (12) 99 (24)	0.38
Recipient's Factors	Induction Immunosuppression (%) Alemtuzumab Anti‐thymocyte globulin Basiliximab	13 (15) 67 (76) 9 (10)	57 (14) 297 (73) 55(13)	0.42
Recipient's Factors	Pre‐emptive transplant (%)	18 (20)	46 (11)	0.03
Recipient's Factors	Mean wait time in days for transplant (SD)	996 (709)	1399 (1049)	< 0.001
Recipient's Factors	DGF in all patients (%)	37 (41.6)	145 (35.5)	0.28
Recipient's Factors	DGF in non‐preemptive (%)	36 of 71 (50.1)	144 of 363 (39.7)	0.09
Recipient's Factors	Length of stay after index transplant (days) (SD)	6.8 (11.3)	6.1 (6.9)	0.59

Most donor, recipient, and immunological characteristics were comparable between recipients of AOOS and non‐AOOS classified kidneys (Table 1). AOOS donors were less likely to have had a cerebrovascular event as a cause of death. AOOS kidneys had higher terminal serum creatinine (1.6 ± 1.2 mg/dl vs. 1.2 ± 1.2 mg/dl, *p* = 0.03) and longer cold ischemia time (22.9 ± 5.5 h vs. 20.4 ± 5.7 h, *p* = 0.003). However, mean KDPI, the proportion of kidneys with KDPI > 85%, donor age, and DCD status did not significantly differ between groups.

Recipients of AOOS kidneys were older (58.9 ± 12.7 years vs. 54.8 ± 13.2 years, *p* = 0.007), more likely to receive preemptive transplants (20% vs. 11%, *p* = 0.03), and had shorter wait times (996 ± 709 days vs. 1399 ± 1049 days, *p* < 0.001) compared with recipients of non‐AOOS kidneys. Recipients of AOOS kidneys were less likely to have cPRA > 20% (10% vs. 21%, *p* = 0.03) or receive retransplants (6% vs. 16%, *p* = 0.007). There was no difference in sex or race between the two groups.

**FIGURE 1:**
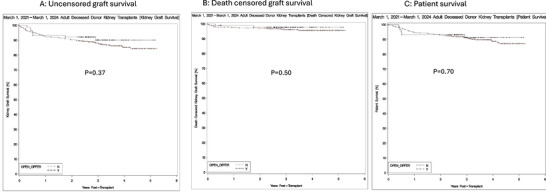
Graft and patient survival. No significant differences in uncensored graft survival (*p *= 0.37), death‐censored graft survival (*p *= 0.50), or patient survival (*p *= 0.70) between the AOOS and non‐AOOS groups.

There were no statistically significant differences between groups in patient survival, graft survival, or death‐censored graft survival at 1, 2, 3, and 4 years post‐transplant (Table 2), as determined by Kaplan‐Meier survival analysis (Figure 1). AOOS transplantation was not associated with increased or decreased risk for graft survival in univariate or multivariate analysis. AOOS transplantation was also not associated with increased or decreased risk for death‐censored graft survival or patient survival in univariate analysis.

**TABLE 2:** Graft and patient survival.

		AOOS	Non‐AOOS	*p*
Graft survival	1 year	93.3%	92.7%	0.37
Graft survival	2 years	92.1%	90.2%	0.37
Graft survival	3 years	90.1%	88.0%	0.37
Graft survival	4 years	90.1%	85.2%	
Death‐censored graft survival	1 year	98.9%	97.5%	0.49
Death‐censored graft survival	2 years	97.7%	97.0%	0.49
Death‐censored graft survival	3 years	97.7%	96.1%	0.49
Death‐censored graft survival	4 years	97.7%	95.6%	
Patient survival	1 year	93.3%	94.9%	0.70
Patient survival	2 years	93.3%	92.9 %	0.70
Patient survival	3 years	91.3%	91.5%	0.70
Patient survival	4 years	91.3%	88.7%	

**FIGURE 2:**
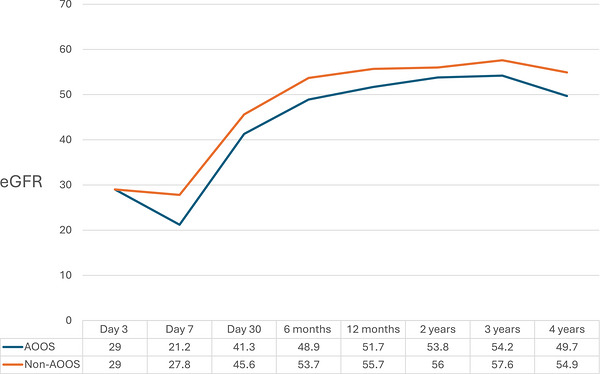
Changes in eGFR at different post‐transplant intervals between the AOOS and non‐AOOS groups.

When graft function was assessed by serum creatinine and eGFR, there were no statistically significant differences between groups at postoperative day 30, 6 months, 12 months, 3 years, or 4 years (Table 3). The trend of eGFR between groups is summarized in Figure 2.

**TABLE 3:** Graft function over time after transplant among recipients of AOOS and non‐AOOS kidneys.

Time post‐transplant	AOOS serum creatinine (mg/dl), mean (SD)	Non‐AOOS serum creatinine (mg/dl), mean (SD)	*p*	AOOS eGFR (ml/min/1.73 m^2^), mean (SD)	Non‐AOOS eGFR (ml/min/1.73 m^2^), mean (SD)	*p*
Days 3	4.26 (2.7)	4.46 (3.16)	0.73	29.0 (.)	29.0 (28.1)	0.99
Days 7	5.32 (3.06)	4.37 (3.22)	0.03	21.2 (22.6)	27.8 (22.9)	0.045
Days 30	2.05 (0.98)	1.91 (1.08)	0.27	41.3 (21.2)	45.6 (21.8)	0.10
6 months	1.70 (0.84)	1.56 (1.08)	0.20	48.9 (18.7)	53.7 (20.9)	0.055
12 months	1.61 (0.59)	1.50 (0.58)	0.12	51.6 (19.5)	55.7 (21.2)	0.10
2 years	1.58 (0.61)	1.49 (0.63)	0.28	53.8 (23.1)	56.0 (20.8)	0.40
3 years	1.43 (0.43)	1.45 (0.64)	0.76	54.2 (18.7)	57.6 (21.8)	0.34
4 years	1.71 (0.60)	1.47 (0.49)	0.16	49.7 (21.5)	54.9 (19.4)	0.44
Average over 30 days	3.47 (1.87)	3.02 (1.93)	0.046	31.0 (20.9)	38.6 (22.2)	0.004
Average over 1 year	2.15 (0.99)	1.95 (0.90)	0.06	44.8 (17.3)	49.6 (19.3)	0.03

AOOS transplantation was not associated with risk for DGF (OR 1.30, 95% CI 0.81–2.07, *p* = 0.28) in univariate logistic regression analysis, nor in multivariate analysis (aOR 1.34, 95% CI 0.82–2.20, *p* = 0.24; Table 4). Likewise, there was no statistical difference in the rate of DGF between groups when including the entire cohort or after excluding preemptive transplants. The mean duration of DGF among the entire cohort was 11.6 ± 15.7 days. The mean duration of DGF among AOOS kidney recipients was 13.5 ± 18.7 days compared with 11.1 ± 14.9 days for non‐AOOS kidney recipients (*p* = 0.39). There was also no significant difference in the number of dialysis sessions between groups, with a median of three sessions (IQR 2.0–5.25) among AOOS recipients compared with three sessions (IQR 2.0–5.0) among non‐AOOS recipients (*p* = 0.91). Among recipients with DGF, 7 (18.9%) of 37 AOOS recipients and 27 (18.6%) of 145 non‐AOOS recipients (*p* = 0.97) had DGF duration ≥15 days. AOOS allocation was not associated with kidney graft survival, death‐censored graft survival, or patient survival (Table 4).

**TABLE 4:** Risk for complications.

		Unadjusted	Unadjusted	Unadjusted	Adjusted	Adjusted	Adjusted
Complications		HR /OR	95% CI	*p*‐value	HR /OR	95% CI	*p*‐value
DGF	Non‐out‐of‐sequence	Ref	Ref	Ref	Ref	Ref	Ref
DGF	Allocation out of sequence	1.30	0.81–2.07	0.28	1.34	0.82–2.20	0.24
Graft survival	Non‐out‐of‐sequence	Ref	Ref	Ref	Ref	Ref	Ref
Graft survival	Allocation out of sequence	0.71	0.34–1.50	0.37	0.72	0.34–1.54	0.40
Death‐censored graft survival	Non‐out‐of‐sequence	Ref	Ref	Ref	Ref	Ref	Ref
Death‐censored graft survival	Allocation out of sequence	0.60	0.14–2.62	0.50	0.61	0.14–2.74	0.52
Patient survival	Non‐out‐of‐sequence	Ref	Ref	Ref	Ref	Ref	Ref
Patient survival	Allocation out of sequence	0.86	0.38–1.91	0.70	0.85	0.38–1.94	0.71

## 4 Discussion

This single‐center retrospective study describes recipient selection and outcomes after AOOS kidney transplantation during the KAS250 era. The principal finding is that, at our center, AOOS kidneys accounted for a modest proportion of deceased donor kidney transplants and were associated with distinct recipient selection patterns. Recipients selected for kidneys categorized as AOOS were older; less sensitized, less likely to be retransplant recipients, more likely to undergo preemptive transplantation, and had shorter waiting times. Despite these differences in recipient selection, patient survival, graft survival, death‐censored graft survival, graft function, and DGF outcomes were comparable with those observed after non‐AOOS kidney transplantation.

An important interpretation of these findings is that AOOS status should not be assumed to represent a uniform donor phenotype or a consistently marginal kidney group. In our cohort, AOOS donors were broadly similar to non‐AOOS donors with respect to age, KDPI, proportion with KDPI > 85%, and DCD status. The main donor and preservation differences were higher terminal serum creatinine and longer cold ischemia time among AOOS kidneys. These differences suggest some degree of functional or logistical complexity, but they were not extreme. Therefore, this study is best understood as an analysis of recipient selection and outcomes after AOOS allocation at one institution, rather than as a study of markedly marginal or extended‐criteria donor kidneys.

The recipient selection pattern observed in this cohort was clinically distinct. Older recipients may require less projected graft longevity than younger recipients and may derive substantial benefit from timely transplantation even when donor kidney durability is uncertain [17, 18, 19]. Lower immunologic risk and avoidance of retransplant recipients may also reduce the risk of early immunologic complications when allocation decisions must be made quickly. At our center, these patterns reflect an attempt to match kidneys entering accelerated placement to recipients perceived to have an acceptable risk‐benefit profile. However, this interpretation is made cautiously because this retrospective study design cannot claim that prospective clinical decision parameters were consistently applied for each individual offer or recipient selection decision.

Our findings share similarities with, and differ from, national analyses of AOOS practice. Liyanage et al. reported contemporary national patterns of out‐of‐sequence allocation and raised concerns regarding potential disparities in recipient selection [8]. In our single‐center cohort, AOOS recipients were similarly older and more likely to undergo preemptive transplantation, but we did not observe significant differences in recipient sex or race between AOOS and non‐AOOS recipients. These differences emphasize that single‐center results should not be generalized to national AOOS practice. Rather, center‐level studies may be useful for understanding how recipient selection and local practice environments interact with a broader allocation process.

The outcome findings are reassuring within the limits of this dataset. Although AOOS kidneys had higher terminal creatinine and longer cold ischemia time, AOOS transplantation was not associated with inferior patient survival, graft survival, death‐censored graft survival, graft function, or DGF outcomes. Jadlowiec et al. similarly reported acceptable outcomes after expedited allocation, although their cohort demonstrated a higher DGF rate [20]. The absence of inferior outcomes in our study may reflect a combination of recipient selection, institutional experience with kidneys at risk for delayed function, and dedicated outpatient DGF management [16]. These findings should not be interpreted to mean that AOOS allocation is uniformly safe across centers or kidney phenotypes, but they do suggest that acceptable outcomes can be achieved in a center‐specific recipient selection framework.

These data have policy relevance because AOOS allocation remains controversial. A strictly sequential match‐run process preserves procedural equity, but it may become inefficient when time‐sensitive placement threatens organ utilization. Conversely, accelerated allocation can improve placement efficiency but may undermine public trust if the process lacks transparency and/or inferior outcomes. The present study does not attempt to resolve that tension. Furthermore, AOOS is regarded as an allocation category observed in clinical practice and not an ongoing transplant practice or endorsement of allocation outside the match run. Instead, it highlights that structured accelerated allocation pathways need to include mechanisms to monitor recipient outcomes and equity, as well as standardized mechanisms of why kidneys enter OOS (or “accelerated”, or “rescue”) allocation.

This study has several strengths, including detailed single‐center characterization of donor and recipient features, assessment of graft function and DGF, and long‐term‐term follow‐up extending to four years. The study also includes a rural‐serving transplant program, which is relevant because geography and transport logistics may influence organ placement in the KAS250 era. However, several limitations merit emphasis. First, this is a single‐center retrospective study, and the findings may not be representative of other transplant centers in similar or other regions. Second, the modest sample size limits statistical power and constrains multivariable modeling for some outcomes. Third, recipient selection bias is inherent in any retrospective comparison of AOOS and non‐AOOS recipients.

A principal limitation is that standardized information describing why individual kidneys entered AOOS allocation was not available. Consequently, we could not determine the relative contribution of donor biopsy findings, machine perfusion characteristics, anatomical complexity, logistical constraints, number of prior declines, refusal reasons, or other center‐ and OPO‐specific considerations. This limitation directly affects interpretation of the donor phenotype. Although AOOS kidneys in our cohort had higher terminal creatinine and longer cold ischemia time, the available data does not support characterizing the AOOS group as uniformly marginal or hard to place.

Future research and allocation policy would be strengthened by mandatory capture of the factors leading to AOOS or accelerated placement and should incorporate multicenter analyses across diverse geographic and practice settings to better characterize variation in AOOS utilization, recipient selection, and outcomes. In addition, predictive models using real‐time offer and donor data may eventually help identify kidneys at risk of nonuse earlier in the allocation process while preserving transparency and equity.

In conclusion, AOOS kidney transplantation at our center represented a modest component of deceased donor kidney utilization during the KAS250 era. Recipients associated with the AOOS classification differed from recipients of non‐AOOS classified kidneys, primarily by older age, lower immunologic risk, more frequent preemptive transplantation, fewer retransplants, and shorter waiting times. Within this single‐center recipient selection distinction, long‐term patient and graft outcomes were comparable with non‐AOOS transplantation, and no significant sex or race imbalance was observed. These findings should not be interpreted as representative of AOOS practices nationally or as evidence that all AOOS kidneys are marginal. Larger studies with standardized reporting of the reasons kidneys enter AOOS allocation are needed to inform transparent accelerated allocation pathways that balance equity, efficiency, and organ utilization. It is also important that the allocation process maintain candidate‐centered allocation and include routine equity monitoring. The pathway also needs to demonstrate optimal outcomes. Such a pathway should use objective and consistent parameters to determine if the process of allocating a donor kidney requires a rescue pathway to avoid non‐use.  Future states of kidney allocation will benefit from transparent national allocation policies that achieves timely placement and reduced discards without reliance on out‐of‐sequence allocation.  It will also require a local clinical component at the transplant center for proper recipient selection. This is an under‐evaluated component.  It is hoped the current report will lend some insights into it.

## Author Contributions

**Sandesh Parajuli:** design, literature review, analysis, manuscript preparation. **Glen E. Leverson:** data analysis, editing. **Didier Mandelbrot:** editing. **Dixon B. Kaufman:** concept, manuscript preparation, literature review, editing.

## Funding

The authors have nothing to report.

## Conflicts of Interest

The authors declare no conflicts of interest.

## Data Availability

The data that support the findings of this study are available on request from the corresponding author. The data are not publicly available due to privacy or ethical restrictions.
